# Arctic climate change and pollution impact little auk foraging and fitness across a decade

**DOI:** 10.1038/s41598-018-38042-z

**Published:** 2019-01-31

**Authors:** Françoise Amélineau, David Grémillet, Ann M. A. Harding, Wojciech Walkusz, Rémi Choquet, Jérôme Fort

**Affiliations:** 1 Centre d’Ecologie Fonctionnelle et Evolutive (CEFE) UMR 5175, CNRS – Université de Montpellier – Université Paul-Valéry Montpellier – EPHE, Montpellier, France; 2 Littoral Environnement et Sociétés (LIENSs), UMR 7266 CNRS - Université de La Rochelle, La Rochelle, France; 3 Environmental Science Department, Alaska Pacific University, Anchorage, AK USA; 4 Freshwater Institute, Fisheries and Oceans Canada, 501 University Crescent, Winnipeg, MB Canada; 5 Institute of Oceanology, Polish Academy of Sciences, Sopot, Poland; 6 Percy FitzPatrick Institute and DST/NRF Excellence Centre at the University of Cape Town, Rondebosch, South Africa

## Abstract

Ongoing global changes apply drastic environmental forcing onto Arctic marine ecosystems, particularly through ocean warming, sea-ice shrinkage and enhanced pollution. To test impacts on arctic marine ecological functioning, we used a 12-year integrative study of little auks (*Alle alle*), the most abundant seabird in the Atlantic Arctic. We monitored the foraging ecology, reproduction, survival and body condition of breeding birds, and we tested linkages between these biological variables and a set of environmental parameters including sea-ice concentration (SIC) and mercury contamination. Little auks showed substantial plasticity in response to SIC, with deeper and longer dives but less time spent underwater and more time flying when SIC decreased. Their diet also contained less lipid-rich ice-associated prey when SIC decreased. Further, in contrast to former studies conducted at the annual scale, little auk fitness proxies were impacted by environmental changes: Adult body condition and chick growth rate were negatively linked to SIC and mercury contamination. However, no trend was found for adult survival despite high inter-annual variability. Our results suggest that potential benefits of milder climatic conditions in East Greenland may be offset by increasing pollution in the Arctic. Overall, our study stresses the importance of long-term studies integrating ecology and ecotoxicology.

## Introduction

Arctic biotas are facing rapid environmental modifications. The Arctic is warming twice as fast as any other place on earth, with visible negative impacts on sea-ice distribution, significantly changing wind regimes and precipitation levels^1,2^. The concurrent emergence of competitors^3^, parasites^4^ or pollutants^5,6^ poses additional new threats for Arctic wildlife^7^. In this context, there is an urgent need for long-term monitoring programs to investigate Arctic species and ecosystem reactions to multiple environmental modifications^8–12^. This is especially true in the North as arctic biomes are poorly studied compared to the rest of the world^7^. In this region, coastal ecosystems deserve particular attention^11^; they host endemic seabirds which have been identified as powerful ecological indicators, and are emblematic for Arctic peoples^9^. Seabirds are subjected to a fair number of long-term monitoring programs in polar regions^8,13–18^. Yet most of these studies focus on the sub-Antarctic and the Antarctic, and integrative, long-term studies of the impacts of environmental changes on the foraging ecology and fitness proxies of arctic seabirds are rare (e.g.^8,15,17,19^). Those are however needed to fully apprehend the incidence of ecosystem modifications on these vulnerable species.

In this study, we focused on little auks (*Alle alle*), the most abundant seabird breeding in the Arctic (estimated 40 to 80 million individuals^20^). Little auks are ecologically important in regional food webs^21^ and could be negatively impacted by ongoing environmental change^22–24^. Notably, little auks are zooplanktivorous, and the distribution of their main prey, Calanoid copepods, is changing along with the warming of the North Atlantic. As a result, the range of the smallest and less energy-rich species, *Calanus finmarchicus*, of Atlantic origin, is extending northwards^25^. It may result in replacement of the two larger and energetically favoured Arctic species, *C*. *glacialis* and *C*. *hyperboreus*, that are preferred by little auks^26,27^. Such a change could prevent birds from covering their energetic needs^22,28^. Although previous studies highlighted that little auks from different colonies can demonstrate strong behavioural plasticity to foraging conditions and prey availability, these studies were performed at the scale of one or a few breeding seasons^29,30^ and longer term impacts are unknown. Furthermore, the largest little auk populations rely on sea-ice and polynya^31^, which are likely to disappear soon from their summer foraging grounds according to IPCC predictions^1^, and this could further modify bird foraging behaviour and reproduction^32^. Similarly, changes in wind regimes could directly affect little auk energetics^33^ and their capacity to respond to the aforementioned changes. At a broader spatial scale, the North Atlantic Oscillation (NAO) index reflects climatic conditions and is commonly used to test for the effects of climate on seabirds (e.g.^34,35^). The NAO index seems to be particularly well suited to studying the population dynamics of migrants that rely on climatic clues^36^. As an example, survival of little auks breeding in Spitsbergen was linked to winter NAO with a time lag of 2 years, with negative effects on the birds being possibly mediated through varying intakes of little auk prey^23^.

In addition to these climatic and resource modifications, little auks could face large changes in the contamination of their environment. For instance, mercury (Hg) concentrations measured in little auks from East Greenland and reflecting the contamination of their environment have increased by 3.4% per year over the last decade^37^. Mercury is a powerful neurotoxin as well as an endocrine disruptor^38^ which could therefore have significant impacts on the reproduction of this arctic seabird species^39,40^. High mercury concentrations could also act as an additional stress factor for adult birds and, in combination with other aforementioned environmental changes, indirectly impact their body condition or foraging performances^41^.

In this context, we propose to examine the multiannual behavioural plasticity of this species in response to environmental change and to investigate impacts on bird fitness. More specifically, and based on recent findings for little auks and their prey, we tested the following hypotheses: (1) the proportion of ice-associated prey in little auk chick diet is decreasing with decreasing sea-ice extent, and the proportion of *Calanus finmarchicus* is increasing^25^. (2) Adult little auks modify their foraging behaviour to cope with a changing environment during summer, to maintain their body condition and the provisioning of their chicks, thereby also maintaining chick growth rates^29,42^. (3) Increasing Hg contamination of little auk environments directly impacts their breeding performances (hatching date, chick growth rate)^39,40^ and acts as an additional stress factor for adults, affecting their body condition^41^. (4) Little auk inter-annual survival is impacted by environmental conditions, both during the breeding and the inter-breeding seasons^23,43^.

To test these hypotheses, we used the longest integrative time-series currently available with respect to little auk ecology and ecotoxicology, which we collected at the breeding colony of Ukaleqarteq (East Greenland, Supplementary Fig. S1) during 12 consecutive summers (2004–2015). We investigated adult foraging behaviour, adult and chick diet, adult winter survival, chick growth, hatching date, and tested the incidence of environmental conditions (sea-surface temperature, sea-ice concentration (SIC), wind force, North Atlantic Oscillation and Hg concentrations inferred from levels measured in bird feathers).

## Results

A summary of sample sizes and biological parameters monitored annually is presented in Table 1. Despite a 12-year long dataset, we were limited by gaps in some of our biological measures and environmental variables (Table 1). This prevented the use of a global approach with causal inference, such as path analysis^44^, as well as the use of principal component analysis to reduce the number of environmental covariables^45^. For this reason, we decided to apply a hypothesis-based approach to target specific questions. We therefore built independent regression models for each biological parameter studied, to test the effects of one or two environmental covariates at a time, to avoid overfitting^45^. A summary of the results is presented in Table 2.

**Table 1:** Sample sizes for each biological parameter.

	2004	2005	2006	2007	2008	2009	2010	2011	2012	2013	2014	2015
Foraging behaviour	4	—	—	6	10	—	—	—	8	8	11	20
Chick diet	35	35	48	39	—	21	20	20	20	20	20	20
Chick growth	—	30	38	29	—	—	12	33	21	29	28	32
Adult body condition	89	502	174	308	85	145	169	143	85	86	129	76
Birds ringed in survival plot	—	191	—	—	—	17	45	30	—	—	50	—
Hg Winter	—	—	—	20	20	20	40	25	20	20	20	20
Hg Summer	—	—	10	12	9	18	8	10	20	—	—	10
Stable isotopes (blood)	—	15	—	15	15	15	15	15	15	15	15	32

**Table 2:** Summary of the interactions found between biological and environmental parameters.

				Environmental parameters						
				summer		Winter			NAO	Time
				↘ SIC	↗ Hg	SST	Wind	Hg		
Biological parameters	Diet	Chick diet	*Apherusa glacialis*	↘	—	—	—	—	—	—
			*Calanus hyperboreus*	NS	—	—	—	—	—	—
			*Calanus glacialis*	↗	—	—	—	—	—	—
			*Calanus finmarchicus*	↘	—	—	—	—	—	—
		Adult diet	δ^15^N	NS	—	—	—	—	—	↗
			δ^13^C	NS	—	—	—	—	—	↘
	Foraging behaviour		Maximum depth	↗	—	—	—	—	—	—
			Dive duration	↗	—	—	—	—	—	—
			Number of dives/day	↘	—	—	—	—	—	—
			Time spent underwater	↘	—	—	—	—	—	—
			Foraging trip duration	NS	—	—	—	—	—	—
	Fitness proxies		Adult body condition	↗	↘	—	—	—	—	—
			Chick growth rate	↗	↘	—	—	—	—	↘*
			Hatching date	↘	—	—	—	NS	—	↗
			Survival probability	NS	NS	NS	NS	NS	NS	NS

Results are presented in the direction of expected environmental changes, with a decrease in sea-ice concentration (SIC) and an increase in mercury (Hg). ↗: increase, ↘: decrease, NS: non significant, —: not tested according to our hypotheses. (*p = 0.052).

### Environmental variables

**Figure 1:**
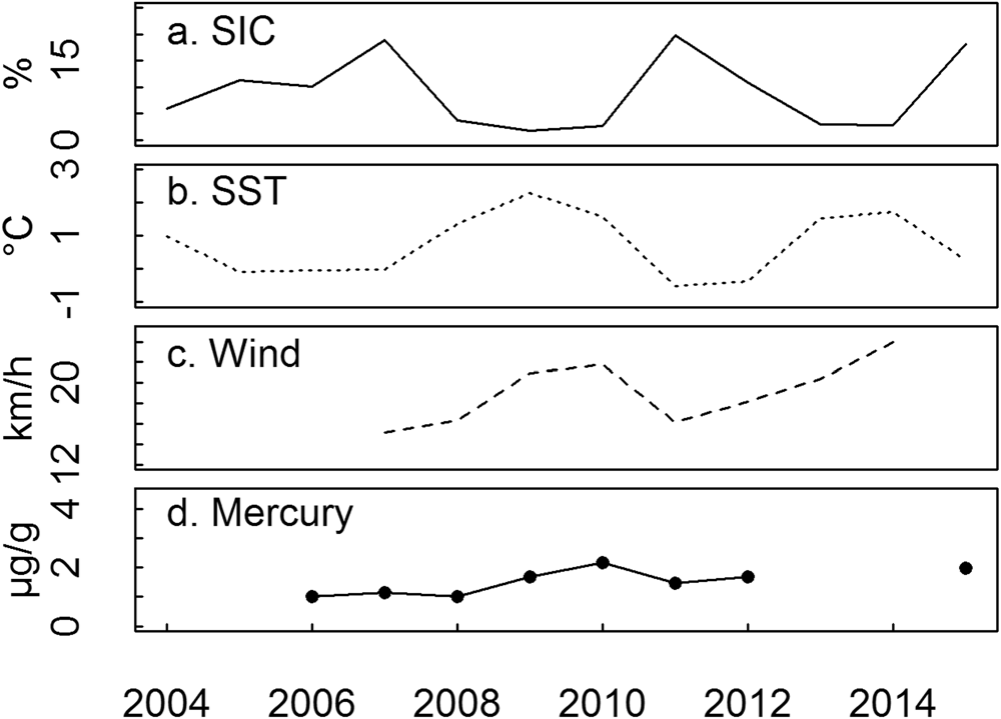
Environmental parameters during the breeding season: (**a**) Sea-ice concentration (SIC, solid line), (**b**) Sea surface temperature (SST, dotted line) and (**c**) wind speed (dashed line) during summer in the vicinity of the studied colony, from remote sensing data. (**d**) Mean mercury concentration of body feathers representing summer contamination (solid line and black dots). SIC was negatively linked with both SST (n = 12, R² = 0.74, p < 0.001, y = −6.1x + 13.6) and wind speed (n = 8, R² = 0.57, p = 0.03, y = −1.3x + 42.9) but was not linked with summer Hg contamination (n = 8, p > 0.1).

Over the period 2004–2015, mean summer SIC varied between 1.8 and 19.8% within the foraging range of little auks (Fig. 1), allowing us to study the links between ice conditions and little auk ecology. SIC was negatively related to sea-surface temperature (SST, n = 12, R² = 0.74, p < 0.001, y = −6.1x + 13.6) and to wind speed (n = 8, R² = 0.57, p = 0.03, y = −1.3x + 42.9, Fig. 1). No temporal trend was found for summer SIC during the study period, however, a decrease in SIC was found in the same area for the period 1979–2014^26^. In addition, the range of SIC encountered in our study period was lower than the range of SIC for the period 1979–2000 (10 to 40%^26^). Summer Hg contamination of little auk environment (derived from body feathers, BF, see methods for details) did not vary with SIC (p > 0.1).

### Variations in chick and adult diet

**Figure 2:**
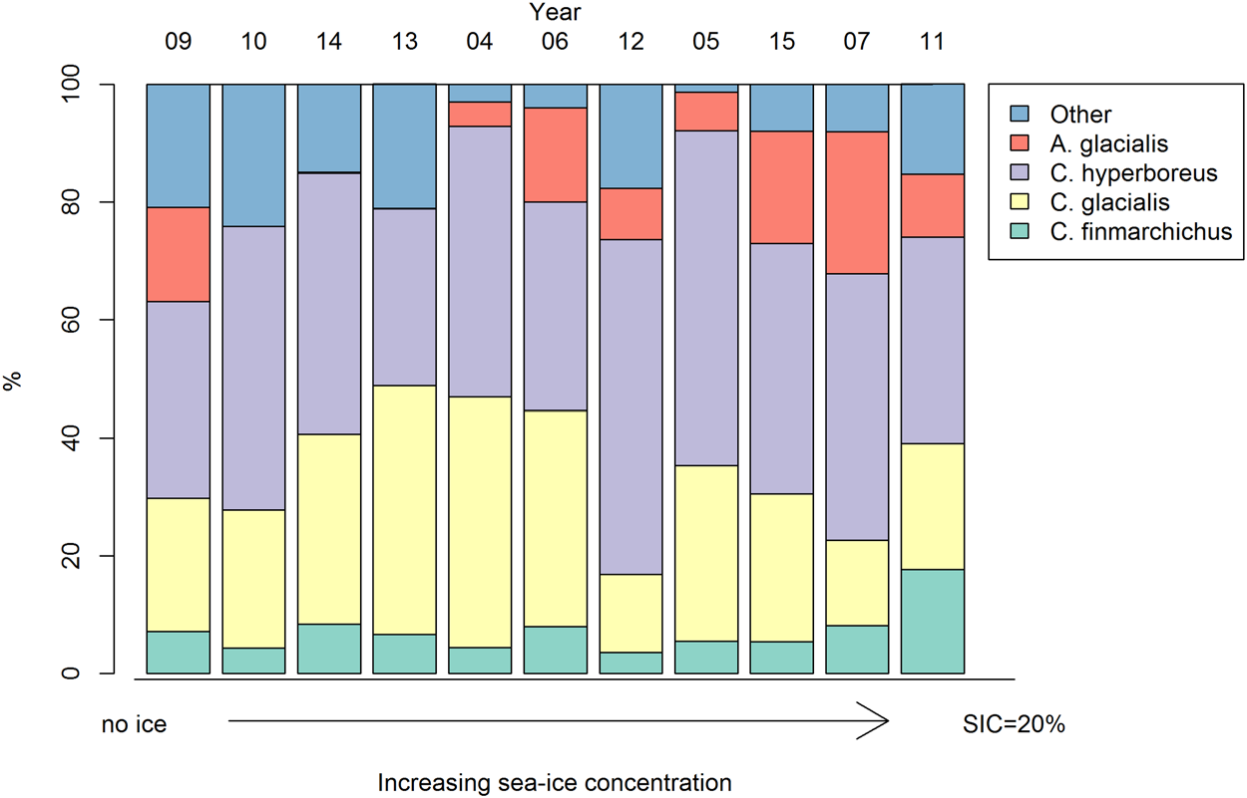
Relative chick diet composition of main prey, classified by increasing sea-ice concentration.

**Figure 3:**
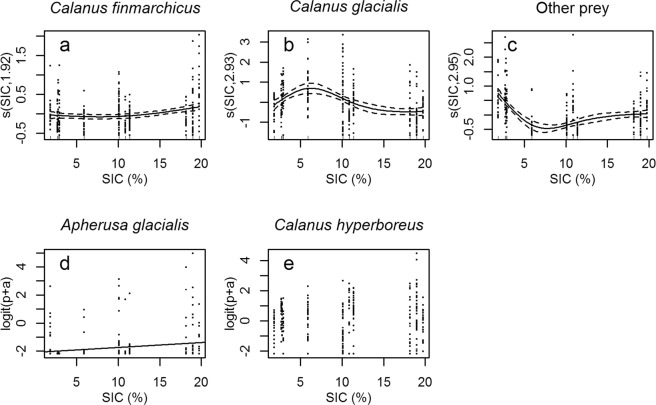
Changes in chick diet with sea-ice concentration (SIC; N = 298). (**a**) *Calanus finmarchicus*, GAM, p = 0.001, R² = 0.05; (**b**) *C*. *glacialis*, GAM, p < 0.0001, R² = 0.13; (**c**) other prey species, GAM, p < 0.0001, R² = 0.28; (**d**) *Apherusa glacialis*, linear model, p = 0.01, R² = 0.04, y = 0.035x-2.1; and (**e**) *C*. *hyperboreus*, linear model, p = 0.25, R² = 0.005. Logit (p + a) represents the logit transformation applied to the proportion of prey (p) and a constant *a* (see Material and Methods for details).

We found an inter-annual variability in chick diet (Figs 2 and 3, n = 298). Changes in the proportions of *Calanus finmarchicus*, *C*. *glacialis* and other prey were best explained by GAMs (Fig. 3a–c) while proportions of *Apherusa glacialis* and *C*. *hyperboreus* were best explained by linear models (Fig. 3d,e). The proportion of *Calanus finmarchicus* increased at higher SIC (N = 298, p = 0.001, R² = 0.05, Fig. 3a). The proportion of *Calanus glacialis* was lower when SIC was >10% (N = 298, p < 0.0001, R² = 0.13, Fig. 3b). The proportion of *Apherusa glacialis* increased with SIC (N = 298, p = 0.01, R² = 0.04, y = 0.035x − 2.1, Fig. 3d). The proportion of *C*. *hyperboreus* did not vary with SIC (N = 298, p = 0.25, R² = 0.005, Fig. 3e). The proportion of other prey was higher when SIC was <5% (N = 298, p < 0.0001, R² = 0.28, Fig. 3c).

**Figure 4:**
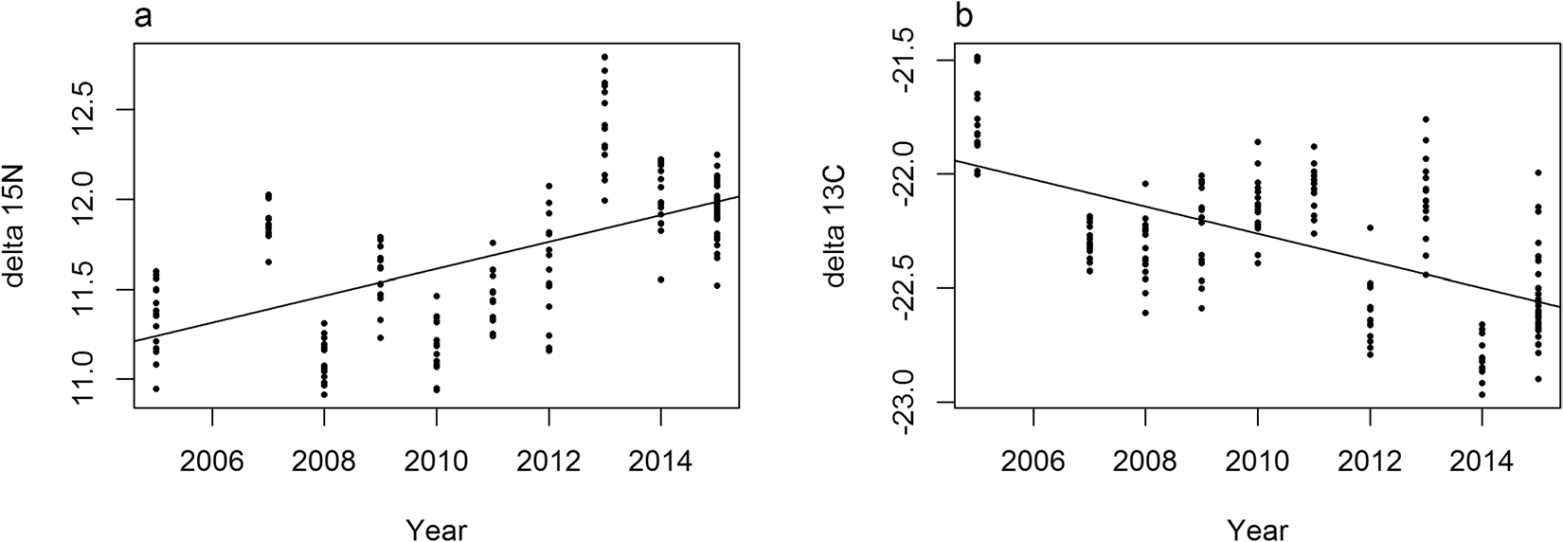
Temporal variation of adult δ^15^N and δ^13^C values (‰) over the study period. (**a**) δ^15^N, linear model, n = 167, R^2^ = 0.32, p < 0.001, y = −138 + 0.07x. (**b**) δ^13^C, linear model, n = 167, R^2^ = 0.36, p < 0.001, y = 96.4 − 0.06x.

Adult diet, as reflected by δ^15^N isotopic values, did not change along with SIC (n = 167, p > 0.1). However, adult foraging ecology changed over time (Fig. 4, trophic status δ^15^N: n = 167, R² = 0.32, p < 0.001, y = −138 + 0.07x, feeding habitat δ^13^C: n = 167, R² = 0.36, p < 0.001, y = 96.4 − 0.06x).

### Foraging behaviour

**Figure 5:**
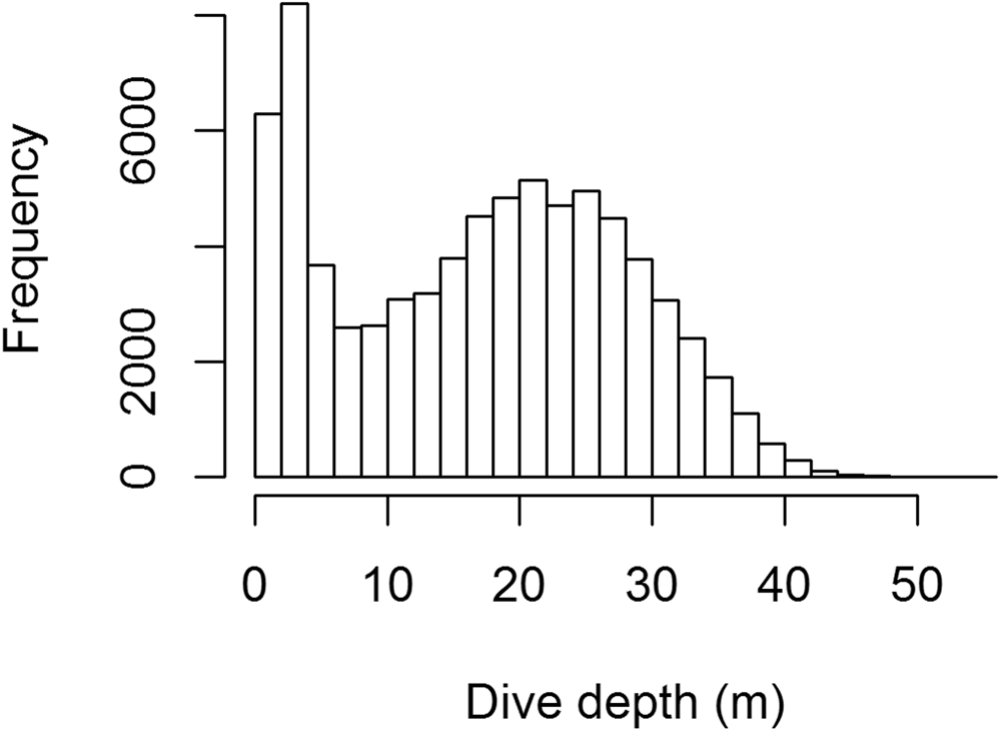
Distribution of maximum dive depths for all recorded dives over the study period. N = 75,173 dives from 67 individuals.

**Figure 6:**
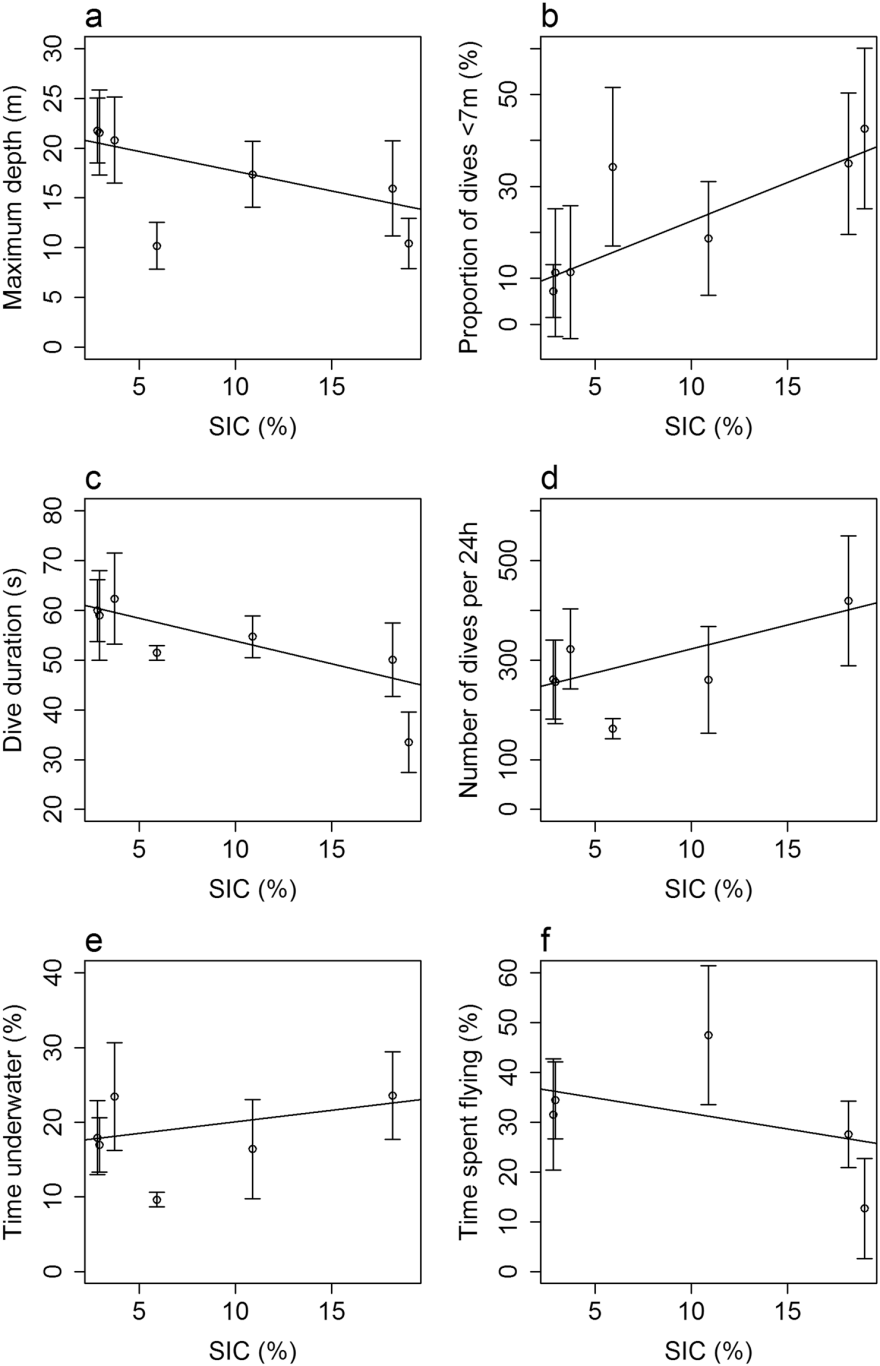
Dive characteristics in relation to sea-ice concentration. (**a**) Maximum depth (n = 67, R² = 0.26, p < 0.001, y = −0.40x + 21.7). (**b**) Proportion of shallow dives (n = 67, R² = 0.40, p < 0.001, y = 1.68x + 5.74). (**c**) Dive duration (n = 67, R² = 0.38, p < 0.001, y = −0.91x + 63.0). (**d**) Number of dives per 24 h (n = 61, R² = 0.26, p < 0.001, y = 9.6x + 226.8). (**e**) Time underwater (n = 61, R² = 0.09, p = 0.02, y = 0.31x + 17.0). (**f**) Time spent flying (n = 53, R² = 0.12, p = 0.01, y = −0.62x + 38.0).

Analysis of dive depths for all dives across the study period showed a bimodal pattern with two distinct peaks: one with dives <7 m and one with dives between 10 and 40 m (Fig. 5). Further investigations showed a link between diving behaviour and SIC (Fig. 6). Dives were deeper and the proportion of shallow dives (<7 m) decreased when there was less ice (Fig. 6a,b; Maximum depth: n = 67, R² = 0.26, p < 0.001, y = −0.40x + 21.7, Proportion of shallow dives: n = 67, R² = 0.40, p < 0.001, y = 1.68x + 5.74). Dives were also longer and birds performed fewer dives per day when SIC decreased (Fig. 6c,d; Dive duration: n = 67, R² = 0.38, p < 0.001, y = −0.91x + 63.0, Number of dives per 24 h: n = 61, R² = 0.26, p < 0.001, y = 9.6x + 226.8). Birds spent slightly less time underwater and more time flying when SIC was low (Fig. 6e,f; Time underwater: n = 61, R² = 0.09, p = 0.02, y = 0.31x + 17.0, Time spent flying: n = 53, R² = 0.12, p = 0.01, y = −0.62x + 38.0). There was no link between foraging trip duration and SIC (n = 53, R² = 0.004, p > 0.1).

### Adult body condition, chick growth and hatching date

**Figure 7:**
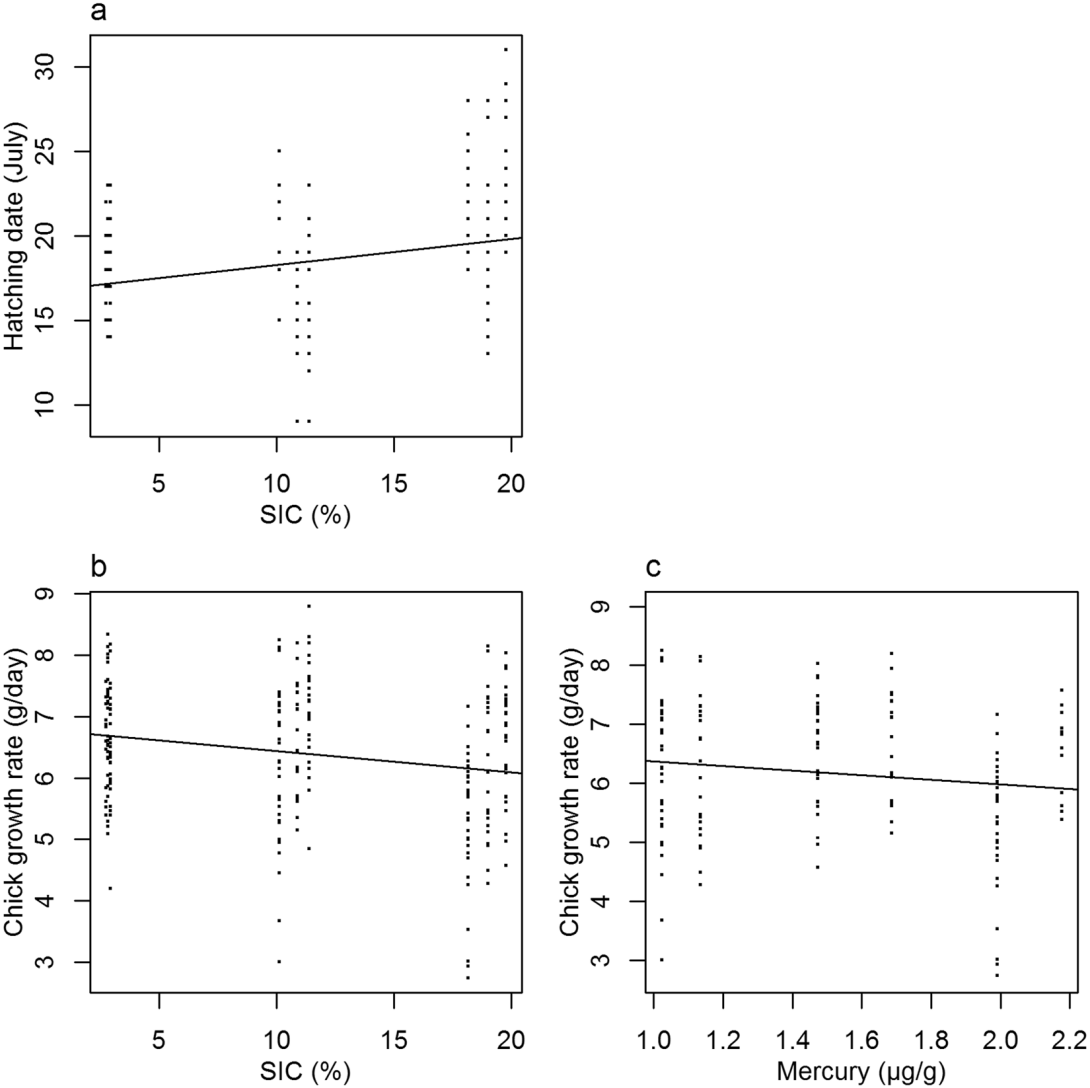
(**a**) Linear relationship between hatching date and SIC (n = 303, R² = 0.08, p < 0.001, y = 16.7 + 0.15x), (**b**) Linear relationship between chick growth rate and SIC (n = 252, R² = 0.03, p = 0.007, y = −0.037x + 6.79), (**c**) Linear relationship between chick growth rate and summer mercury contamination (n = 165, R² = 0.03, p = 0.02, y = – 0.66x + 7.2).

Hatching date was delayed when SIC increased (n = 303, R² = 0.08, p < 0.001, y = 16.7 + 0.15x, Fig. 7a) but was not linked to female exposure to Hg during winter (n = 225, p > 0.1). Chicks hatched later over time (n = 303, R² = 0.11, p < 0.001, y = −660 + 0.34x).

A decrease in chick growth rate (mass gained per day) was observed when SIC increased (n = 252, R² = 0.03, p = 0.007, y = −0.037x + 6.79, Fig. 7b). Chick growth rate decreased when summer Hg increased (n = 165, R² = 0.03, p = 0.02, y = −0.66x + 7.2, Fig. 7c) and chick growth rate tended to decrease over time (n = 252, R² = 0.02, p = 0.052, y = 103–0.05x).

**Figure 8:**
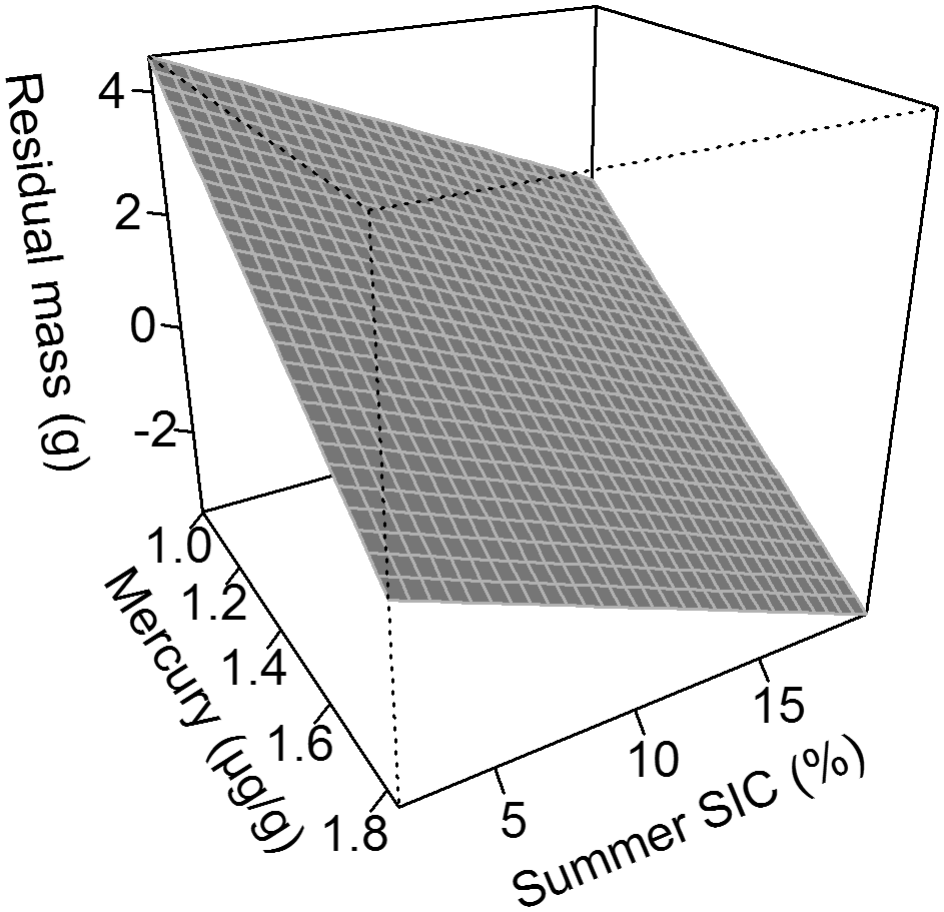
Predicted effects of summer mercury (Hg) contamination and SIC on adult body condition (residual body mass). Predictions were calculated from the model y = −0.030x_1_–4.2x_2_–0.18x_3_ + 9.9, with x_1_ = day of year, x_2_ = Hg, x_3_ = SIC.

Adult body condition worsened with increasing summer Hg and increasing SIC (n = 1051, R² = 0.04, p(Hg) < 0.001, p(SIC) < 0.001, y = 9.9–0.030x_1_–4.2x_2_–0.18x_3_, with x_1_ = day of year, x_2_ = Hg, x_3_ = SIC, Fig. 8), but there was no difference between years (n = 1787, p > 0.1).

### Survival

**Figure 9:**
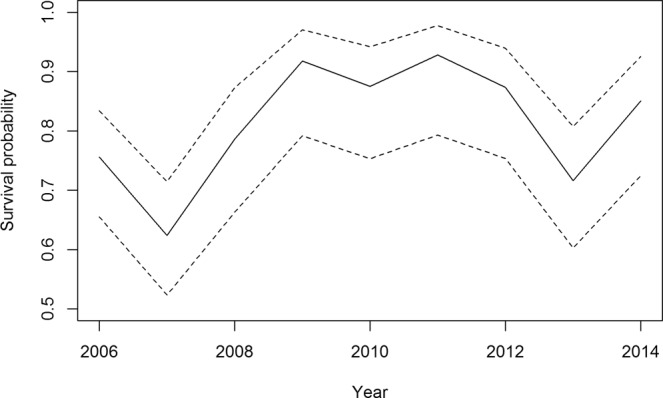
Survival probability of adult little auks over the study period. Year represent the recapture year (e.g. 2015 for the 2014-2015 survival rate). Dashed lines represent 95% CI.

Results of the goodness-of-fit tests are detailed in the methods and in Table 3. A summary of the selection model process is presented in Table 4. We constructed a model with capture heterogeneity due to the physical structure of the colony where some burrow entrances were harder to monitor^46^. Survival probabilities for the best model (φ(t), p(het + t)) is presented in Fig. 9. No direct relationship was found between survival and environmental parameters (tested one by one: North-Atlantic Oscillation (current year, previous year and two years before^23^), SST in their wintering area (current and previous year) and in their breeding area, SIC, wind conditions, summer and winter Hg). Survival probability was lower for two years: 2006–2007 and 2012–2013 (Fig. 9).

**Table 3:** Goodness of fit of the CJS (Cormack Jolly Seber) model by U-CARE. The CJS model is rejected.

Test	df	χ²	P
3.SR	5	9.45	0.092
3.SM	4	5.36	0.252
2.CT	8	77.71	<0.001
2.CL	5	5.10	0.404
Sum	22	97.6	<0.001

**Table 4:** Model selection for the survival analysis.

ϕ	p	Np	ΔQAICc	QAICc W	Deviance
**STEP 1: models with time and capture heterogeneity**					
time	het x time	30	13.26	0.00	1367.80
**time**	**het** + **time**	**22**	**0.00**	**0.87**	**1371.41**
time	het	13	7.35	0.02	1397.42
1	het + time	13	26.84	0.00	1416.91
time	time	19	105.13	0.00	1482.80
time	1	11	128.21	0.00	1522.38
**STEP 2: models with environmental data**					
summer wind	het + time	15	4.61	0.09	1390.56
winter wind	het + time	15	8.27	0.01	1394.22
winter Hg + winter SST	het + time	15	16.85	0.00	1402.81
winter SST n-1	het + time	14	18.04	0.00	1406.05
summer Hg + SIC	het + time	15	18.48	0.00	1404.43
prop. of winter wind > 40 km/h	het + time	15	20.08	0.00	1406.03
SIC	het + time	14	21.28	0.00	1409.29
prop. of winter wind > 30 km/h	het + time	15	23.04	0.00	1408.99
NAO n-1 + winter SST	het + time	14	26.50	0.00	1414.51
NAO n-1	het + time	14	27.25	0.00	1415.26
NAO n-1 + NAO n-2	het + time	14	27.70	0.00	1415.72
NAO n-2	het + time	14	28.26	0.00	1416.27
NAO	het + time	14	28.40	0.00	1416.41
winter SST	het + time	14	28.53	0.00	1416.55
time x summer body condition	het + time	14	28.68	0.00	1416.70
NAO + SST n-1	het + time	14	28.81	0.00	1416.83
winter SST + NAO	het + time	14	28.82	0.00	1416.83
summer Hg + winter Hg	het + time	15	29.60	0.00	1415.55
SST n-1 + NAO n-1	het + time	14	29.91	0.00	1417.92
summer Hg	het + time	15	31.55	0.00	1417.50
winter Hg	het + time	15	33.03	0.00	1418.98
NAO + NAO n-1	het + time	14	33.74	0.00	1421.76
winter SST + winter SST n-1	het + time	14	33.81	0.00	1421.82
summer δ^15^N	het + time	13	363.62	0.00	1753.68
summer δ^13^C	het + time	13	366.85	0.00	1756.91
summer δ^15^N + summer δ^13^C	het + time	13	366.98	0.00	1757.05
summer SST	het + time	13	367.76	0.00	1757.83

ϕ: survival probability. p: resighting probability. Np: number of identifiable parameters. ΔQAICc: difference between the current model AIC and the smallest AIC. QAICc W: QAIC weight. het: heterogeneity of capture (seen with a high probability/seen with a low probability/not seen). NAO: North Atlantic Oscillation winter index. SST or NAO n-1 = the winter of year n-1. δ^13^C and δ^15^N: summer isotopes from blood. The best model is in bold.

## Discussion

Using a unique dataset of biological parameters from a 12-year long-term monitoring program in East Greenland, we found that little auks are impacted by current environmental changes occurring in the Arctic. (1) As expected, the proportion of ice-associated species in chick diet was related to SIC, but the proportion of *C*. *finmarchicus* (copepod of Atlantic origin) did not decrease with increasing SIC. (2) Despite substantial plasticity in foraging behaviour and diet, adult body condition and chick growth rates decreased when SIC increased. (3) Adult body condition and chick growth rate were negatively related to summer levels of mercury. (4) Despite these changes, adult survival was not linked to environmental variables.

### Methodological caveats

Overall, we detected strong environmental impacts on little auks, but our work entails methodological limitations. Indeed, organism answer to overall forcing is the integration of all environmental parameters, and disentangling the relative importance of each factor as well as their interactions requires advanced statistical methods that we could not apply due to temporal gaps in our datasets (Table 1). Consequently, as we used only one or two environmental independent variables at a time to explain one biological parameter with a hypothesis-based approach, the percentage of covariance explained by our significant relationships was low. It is therefore crucial to continue long-term monitoring programs to increase sample sizes. In addition, we possibly missed important additional environmental factors. For instance, we know that the timing of breeding in little auks is linked to the timing of snow melt in spring, which determines nest accessibility^47^. Spring snow melt can be approximated by spring temperature, but we did not have access to this information at our study site. Also, other pollutants could, in addition to Hg, impact little auk reproduction and survival (e.g.^48,49^) but were not considered in the present study. Concerning survival data, we could not take oil spills into account^50^, although it is known that little auks can be highly impacted during winter, with for instance an estimated 22,000 guillemots and little auks killed by hydrocarbon contamination in 2011–2012 off Newfoundland^51^.

### Foraging plasticity as a buffer to climate change

Among all biological parameters investigated, foraging behaviour was the most variable. While little auks foraged in the same areas at the shelf break with or without sea-ice^26^, we found that diving behaviour changed with SIC: Birds performed shallower and shorter dives when SIC was the highest (Fig. 6). Thereby, dives <7 m (Fig. 5, Supplementary Fig. S2) probably reflected foraging directly underneath the sea ice, to feed on sympagic species such as *Apherusa glacialis*, and indeed the proportion of this species in chick diet increased along with SIC (Fig. 3d). This was also supported by preliminary results concerning birds for which diving behaviour and diet were collected simultaneously (n = 15, Amélineau *et al*. unpublished).

Little auks seem capable of switching from pelagic to below-ice feeding, and therefore to cope with a wide range of foraging conditions. Their energy expenditure as determined using the doubly-labelled water (DLW) technique thereby seemed to remain unchanged^29^, yet additional studies combining 3D acceleration recordings of their actual foraging movements, and DLW are needed to fully test the impact of foraging plasticity on energy balance^52–54^.

Changes in little auk diet reflect their preferences, as well as prey availability in the environment. Recent studies suggest that little auks favour larger and fattier species^26,27^ and, therefore, observed changes in prey proportions are likely to reflect the availability of larger prey species in the foraging range of birds. In the North Atlantic Arctic, it is predicted that smaller *C*. *finmarchicus* should be present during “warm” conditions (high SST, low SIC), and larger *C*. *glacialis* and *C*. *hyperboreus* in “cold” conditions (low SST, high SIC)^25,55,56^. However, proportions of little auk main prey, the three species of *Calanus*, did not vary as expected: the proportion of *C*. *finmarchicus* slightly increased at higher SIC, and the proportion of *C*. *glacialis* decreased at higher SIC. Underlying mechanisms driving zooplankton abundance at a given place are complex and do not depend solely on local summer SIC/SST, but might also vary with, for instance salinity and depth^57^ which were not included in this study. However, little auks have access to distant foraging areas spread over dozens of kilometers where depth and salinity and thus prey availability might vary substantially^27^. In addition, it should be noted that although slightly increasing with SIC, the proportion of *C*. *finmarchicus* in chick diet remained low in comparison to other *Calanus* species, whatever the SIC (Fig. 2). Interestingly, despite no clear pattern in chick diet, adult diet shifted to a higher trophic level (higher δ^15^N values) and to more offshore feeding habitats (lower δ^13^C values) during the study period (Fig. 4). Such stable isotope analyses are particularly integrative and could therefore reflect fine changes occurring in the longer term among the zooplankton community. Moreover, it is still unclear whether adults and chicks feed on the exact same prey because the compositions of their diets have never been measured concomitantly. While stomach contents of adults are comparable to gular pouch samples^58,59^, stable isotope studies suggest that there could be a difference^60,61^, as do our observations. More generally, little auks seem able to cope with different prey assemblages in their environment^27,30,62–64^.

### Do little auk living conditions improve in the absence of sea-ice?

Little auks have a non-obligate affinity to sea-ice which varies according to location or timing of the year. During the breeding season, they can forage at the marginal ice zone in Spitsbergen^32,65^ but can also thrive in the absence of sea-ice^26,66^. After the breeding season, birds from different colonies migrate towards higher latitudes and the MIZ to moult^67,68^, before reaching their wintering grounds. At our study site, contrasting SIC from year to year allowed to study impacts on little auk foraging and fitness proxies.

Interestingly, adult body condition and chick growth rates were higher when SIC was low, meaning that less sea-ice and higher SSTs provided better environmental conditions for breeding little auks in East Greenland. The link between environmental conditions and fitness proxies could be direct, as higher temperatures reduce energy requirements for thermoregulation^33^, and energy gained could then be reallocated to body maintenance or chick rearing. Linkages could also be indirect, via trophic interactions and bottom-up effects: lower SIC during summer reflects an earlier sea-ice breakup and a shorter lag between ice-algae bloom and pelagic phytoplankton bloom^69^. Our results suggest that prey quality and/or availability would be better when sea-ice breakup occurs earlier. However, this is not in accordance with previous findings from Spitsbergen, where earlier sea-ice breakup lead to a mismatch between algal blooms and copepod *C*. *glacialis* phenology, and ultimately to a lower chick survival in little auks and Brünnich’s guillemots^70,71^. Changes in the trophic interactions occurring in the Western Greenland Sea are probably more complex and reflects high variability of local conditions throughout the Arctic^12^. Lastly, SIC encountered during the study period (2004–2015, 9.1 ± 6.8%) were already lower than SIC encountered during the period 1979–2003 (20.0 ± 10.4%)^26^ and may already feature suboptimal conditions for little auks.

Regarding SSTs, previous studies suggested that the most profitable foraging areas for little auks are located in the cold waters encountered in the Sørkapp Current (SW Spitsbergen) and in the East Greenland Current that contain bigger *Calanus* species^27,72^. Our results contrast with those from previous studies performed in Spitsbergen where higher SSTs were associated with a higher proportion of *C*. *finmarchicus* in the environment and in chick diet, and led to a lower chick survival or probability to fledge^22,72^, and to a lower adult survival^23^. SSTs are higher in Western Spitsbergen than in East Greenland, and these contrasting findings suggest that little auk fitness could follow a quadratic relationship with SST, peaking at intermediate SST. An increase in SST could then be beneficial at low SSTs (East Greenland, mean SST of 0.67 °C) but detrimental at high SSTs (Spitsbergen, mean SST of 1.81 °C at Hornsund and 4.51 °C at Kongsfjorden). However, little auks did not seem impacted by observed changes in SST around Bjørnøya, where they foraged in warm waters (median SST of 6.6 °C) during the period 2005–2012^66^.

Among pagophilic seabird species, reactions to variations in SIC are diverse and depend on species-specific sea-ice affinity. High SIC during the breeding season can reduce access to prey and lead to lower breeding success for moderately pagophilic seabirds^15,16,73^. Species that are more dependent on sea-ice, on the contrary, have lower breeding success and survival when SIC is reduced, and have to travel over longer distances to reach the MIZ^74,75^. At the larger temporal and spatial scales, changes in SIC likely lead to changes in species range to the detriment of pagophilic species^9,76^.

### Pollutants offset observed benefits from lower SIC

Hg concentrations during summer increased in adult little auks, likely linked to an increase in prey Hg concentrations over time^37^, but also to changes in diet towards prey that are higher in the food chain (biomagnifications)^77^, as reflected by the increase in δ^15^N in adult blood (Fig. 4a). Although measured Hg concentrations were below toxicity thresholds^38,78^, they were negatively related to adult body condition and chick growth rate. These observed effects suggest that Hg might act as a cumulative stressor which, in combination with other environmental constraints like the quality of their habitat or resource availability^41^, could impact the condition of marine predators beyond its single effects. In addition, one should also bear in mind that little auks are exposed not only to Hg, but to a variety of pollutants reaching the Arctic from northern mid-latitude industrial regions, some of them emerging but already of high concern in this sensitive region^79^. We specifically focused on Hg, which is known to bioaccumulate in polar regions and severely impact marine top predators^38,78^, but these other pollutants may as well impact little auk metabolism and ultimately their body condition and growth rate, such as organochlorine pesticides or PFASs^80^. In addition, Hg disrupts breeding behavior in black-legged kittiwakes and snow petrels^40,81^ and this mechanism could also explain reduced chick growth when Hg concentrations are high in our study. However, no link was found between Hg and adult survival in our study, as well as in other seabird species^48,82–84^.

Hg levels in the Arctic are modified by ongoing environmental changes^85^. In particular, increasing Hg trends are expected with permafrost thawing, the warming of ocean water masses and increasing human activities in the Arctic^85–87^. According to our results, negative effects of increasing Hg could offset observed positive effects of climate warming in East Greenland. This stresses again the complexity of biological answers to environmental changes and the need for integrative approaches.

## Conclusion

Understanding how animals will cope with environmental changes is a topical challenge in ecology. Since the Arctic is warming twice as fast as the rest of the world it can be seen as a natural laboratory to anticipate changes occurring at a more global scale. Unfortunately, logistical constraints, including year round access, limit fieldwork studies in this part of the world. In addition, biological responses are complex and integrate environmental constraints which may be logistically difficult to evaluate at remote locations. Our results highlight the importance of pursuing long term monitoring programs in the Arctic to improve dataset length and quality, and gain power to elaborate more complex models^88^.

## Methods

### General fieldwork context

All field work in East Greenland was conducted in accordance with guidelines for the use of animals^89^. Experiments were approved by the Danish Polar Center and the Government of Greenland, Ministry of Environment and Nature and Department of Fisheries, Hunting and Agriculture (No. 512–240 (2005), No. 512-258 (2006), No. 07-501 (2007), No. 66.24/23 (2008), No. 66.01.13 (2009 and 2010), No. 2011-047447 (2011), No. 2012-065815 (2012), No. 2013- 083634 (2013), No. 2014-098814 (2014) No. 2015-115290 (2015).

Little auks from Ukaleqarteq (Kap Höegh, East Greenland, 70°44′N, 21°35′W, Supplementary Fig. S1a) were studied during the breeding season (mid-July/mid-August) from 2004 to 2015. Birds breed under rocks in steep boulder fields, where they raise a single offspring. Adult birds fly out to sea where they forage on zooplankton, which they bring back to their chick in a sublingual pouch. During the inter-breeding period (Sept-May), birds migrate to wintering areas in the North Atlantic, notably off Newfoundland^90^. Each summer, a set of biological parameters detailed below were monitored, and sample sizes are presented in Table 1. Adult birds were caught either in their nest or on the surrounding rocks using a lasso or noose carpets. Breeding status was assessed by the presence of a chick, a full sublingual pouch or a brood patch. Handling time was <10 min. For all sampling except for the survival study (see below), each year different individuals were studied. Therefore, our investigations were mainly conducted at the population – and not at the individual – level.

### Chick and adult diet

Breeding adults were captured on arrival at the colony and the content of their sublingual pouch (chick diet) was removed and stored either in 4% borax-buffered formaldehyde solution (2005 to 2007) or in 70% ethanol (2008 and beyond). Samples were identified to the lowest possible taxonomical level under a stereomicroscope following groups presented in Harding *et al*.^28^. Adult diet was estimated from stable isotope analyses (δ^15^N and δ^13^C) performed on total blood samples^61^. δ^15^N isotopic values reflect the relative trophic position of birds and are considered an indicator of their diet a couple of weeks before the sampling^91^. δ^13^C was also considered as an indicator of bird foraging habitats with higher values representing more coastal habitats^91^. Blood samples (0.3 ml) were collected from bird brachial vein, stored in 70% ethanol and kept frozen at −20 °C. Prior to analyses, blood samples were freeze-dried for 48 h and homogenized. Stable isotope analyses were then performed on ~0.5 mg subsamples of homogenized, non-lipid extracted whole blood loaded into tin cups, and using an elemental analyzer (Flash EA 1112, Thermo Fisher) coupled in continuous flow mode to an isotope ratio mass spectrometer (Delta V Advantage, Thermo Fisher, Bremen, Germany). Stable isotope abundances were expressed in δ notation as the deviation from standards in parts per thousand (‰) according to the equation: δX = [(Rsample/Rstandard) − 1] × 1000 where X is ^13^C or ^15^N and R is the corresponding ratio ^13^C/^12^C or ^15^N/^14^N. Standard values were Vienna Pee Dee Belemnite (VPDB) for C and atmospheric N_2_ (air) for N. Replicate measurements of internal laboratory standards (acetanilide) indicated that the measurement error was <0.2% for both δ^15^N and δ^13^C values.

### Foraging behaviour

Numbers of equipped birds are presented in Table 1. Breeding adults were equipped with temperature-depth recorders (TDRs) attached ventrally, recording at 0.2, 0.5 or 1 Hz for 2–5 d during the chick-rearing period. Details on TDR types and attachment methods are presented in Supplementary Methods and Supplementary Table S1. Data were analyzed with MultiTrace™ to extract maximum dive depth, dive and pause duration for each dive. Depth was corrected for Star Oddi devices because they showed a slight underestimation of depth according to a calibration made in Amélineau *et al*.^26^. We also measured time spent flying and foraging trip duration following Welcker *et al*.^92^, and calculated the time spent underwater, and the number of dives per day. For each parameter, a mean value per individual was calculated and used for statistical analyses.

### Hatching date and chick growth

Nests were controlled for hatching date and chicks were weighed every second day. For each chick, we calculated the chick growth rate (g d^−1^) as the slope of the linear growth period^26^ (4–14 days). Due to logistical constraints usually preventing measurements after August 10, we could not control all nests until fledging to measure fledging success (range of fledging age = 21–31 days^93^).

### Adult body condition and mercury contamination

Each handled adult was weighed (g), and wing and head-bill lengths were measured (mm). We constructed a body condition index, correcting adult body mass by wing length and head-bill length to take bird size into account. The body condition index was calculated as the residual body mass from a regression of body mass on wing length and head-bill length^43^.

Total Hg was measured on one complete back cover feather (abbreviated BF hereafter) or one complete throat feather (abbreviated HF hereafter) using an advanced Hg analyzer spectrophotometer (Altec AMA 254) as described in Bustamante *et al*.^94^. Hg in little auk BF reflect the amount of Hg accumulated by birds during the previous breeding season spent in East Greenland (year preceding the sample), while Hg in HF reflect the amount of Hg accumulated during the previous winter (see Fort *et al*.^37^). Hence, BF collected from 2007 to 2016 were analyzed for comparison with the biological time-series. Previous studies showed that >90% of Hg in seabird feathers is methyl-Hg (Me-Hg), the most toxic form of Hg^95^. Total Hg measured in feathers is thus considered as an indicator of bird exposure to Me-Hg. Hg concentrations measured in bird feathers are also an indicator of the contamination level of the food chain on which birds feed during both seasons^37^. Analyses were repeated two or three times (two or three feathers) for each bird and feather type until the relative standard deviation for two samples was <10%; samples not meeting this criterion were excluded from the analysis. The mean Hg concentrations for those two or three measurements were then considered for statistical analyses. To ensure the accuracy of measurements, a certified reference material (CRM) was used [Lobster Hepatopancreas Tort-2; NRC, Canada; Hg concentration of 0.27 ± 0.06 µg.g^−1^ of dry weight (dw)]. The CRM was measured every 10 samples and the average measured value was 0.26 ± 0.01 µg.g^−1^ dw (n = 113). Additionally, blanks were run at the beginning of each sample set. The detection limit of the method was 0.005 µg.g^−1^ dw.

### Survival analysis

One plot of the colony was dedicated to a capture-mark-recapture experiment. Birds (n = 333) were marked with a unique code composed of 3 colour rings and one metal ring. Each season, recapture sessions lasted 6 d with 7 h of continuous observation per day. Data were analyzed using a capture-recapture model^96^ with E-SURGE. We first built a structural model without any external covariate. To define the structural model, we first did single state goodness-of-fit tests (GOF, Table 3) using U-CARE^97^. Only the 2.CT test was significant, indicating a difference in the probability of being recaptured at i + 1 for birds seen and not seen at occasion i. In order to take into account recapture heterogeneity among marked birds, we used a model with two classes of capture^98^ and defined three states: individuals with a high recapture probability, individuals with a low recapture probability and dead individuals. Changes of state between high and low recapture probability were not permitted. Such a structure explained our data better than a model including trap-dependence or allowing changes of recapture probability through time (if, for example, this was linked to breeding status). Biologically, recapture heterogeneity was due to the structure of the colony, where some birds nest in areas where it is more difficult to see them enter and leave their burrows. Such a model with recapture heterogeneity was used for least auklets (*Aethia pusilla*) that also breed in burrows like little auks^46^. The model selection was conducted with E-SURGE^99^. The general starting model was (φ(t), p(het.t)), where “het” denotes the heterogeneous effect on capture with two levels (seen with a high or low probability), t denotes the time effect. Models were selected based on Akaike^100^ Information Criterion (AIC) corrected for sample sizes and overdispersion (QAICc). During first step, we selected the best model with only time and state as explanatory variables. In step two, we included environmental variables (one or two at a time^45^, the absence of correlation was verified when two environmental variables were included simultaneously) to the model with the best structure in the first step (Table 4).

### Environmental data

For the summer period, environmental data were dealt with within a 160 × 200 km plot surrounding the colony, which also included little auk at-sea habitats as determined through GPS tracking^26^ (Supplementary Fig. S1b). Monthly SST came from the Multi-scale Ultra-high resolution (MUR) SST analysis from the NASA (v4.1, global 0.01° resolution, monthly) and were acquired from http://coastwatch.pfeg.noaa.gov/erddap/index.html. Monthly wind data came from the Metop/ASCAT data set (0.25° resolution, monthly, starting in 2007) from CERSAT and were acquired from http://cersat.ifremer.fr/. Daily sea-ice concentration (SIC, percentage of sea surface covered by ice in a given area) came from the Eumetsat OSI SAF and were acquired from http://osisaf.met.no/, using the Global Sea Ice Concentration reprocessing dataset (0.25° resolution, daily). For wind and SST data, we used monthly values for July and we calculated the mean annual value for the foraging area defined above. For SIC, we calculated a mean annual value from the daily SIC concentration in the area between 15^th^ July and 15^th^ August.

Overwintering locations of birds from Ukaleqarteq were known from birds equipped with geolocators^90^, and we defined the core wintering area of birds as the 50% kernel area of positions between 1^st^ November to 28^th^ February obtained for 94 little auks equipped between 2009 and 2015^101^. Yearly winter environmental conditions (wind speed, SST) were calculated as a mean value within the core wintering area from November to February from monthly values of the datasets mentioned above. In addition, we calculated the proportion of days with high winds (mean daily wind speed > 40 km/h) during the same period, using daily wind speeds from the Metop/ASCAT data set (0.25° resolution). Data for the North Atlantic Oscillation (NAO) came from the UCAR and were acquired from https://climatedataguide.ucar.edu/climate-data. Herein we used the winter NAO index^23^. Winter environmental parameters were used for the survival analyses only.

### Statistical analyses

All analyses were performed with the R software (v. 3.4.2; R core team 2017). We adopted an hypothesis-based approach to study the link between environmental variables and biological parameters, i.e. we tested specific linear models with one or two explanatory variables at a time that are meaningful in a biological context, instead of testing all possible combinations of factors. This ensured to reduce type 1 errors, and to avoid overfitting, as well as issues regarding autocorrelation among environmental variables^45^. In particular, summer SIC, summer SST and summer wind were highly related (Fig. 1). Among these environmental variables, SIC was selected as the main environmental parameter to test, due to its strong and direct influence on the foraging behaviour of little auks at our study site. Mean yearly Hg concentrations in head or body feathers were considered as environmental variables reflecting Hg found in the environment in winter and in the previous summer, respectively^37^. Hg was either tested independently when a direct influence is expected (chick growth rate and hatching date), or tested concomitantly with SIC when it is expected to be an additional stress factor (adult body condition). We also investigated temporal variations of biological parameters.”Day of year” was included in the models with adult body condition as a response variable as body mass is slightly decreasing along the breeding season.

For adult diet, foraging behaviour, adult body condition and chick growth rate, we performed linear regressions to model the relationship between biological variables and environmental variables, when the assumptions were met. We did not use mixed-effects models because each bird was only sampled one time. Results of the tests were considered significant when p-value was <0.05. For changes in prey proportion in chick diet in relation to SIC, we first used Generalized Additive Models (GAMs) as no linear curve was expected. We performed a logit transformation of the proportions (p): log((p + a)/(1 − (p + a)), and where *a* is a constant (*a* = 0.1) in order to get a logit for all proportions including null values. Each GAM was then compared with a linear model and the model with the lower AIC was retained (all differences in AIC were greater than 2).

## Supplementary information

Supplementary information

## Data Availability

All data are available on request from the CEFE CNRS database accessible here: http://www.cefe.cnrs.fr/fr/ressources/base-de-donnees/1114-puechdb-station-experimentale-de-puechabon-tour-a-flux-sp-23356.
