# Supplementary material for: Arctic climate change and pollution impact little auk foraging and fitness across a decade
Source: Sci Rep. 2019 Jan 31;9:1014. doi: 10.1038/s41598-018-38042-z (PMC6355795; doi:10.1038/s41598-018-38042-z)
Supplement: Supplementary file 1 — Supplementary information [file 41598_2018_38042_MOESM1_ESM.pdf]

Supplementary Information

Arctic climate change and pollution impact little auk foraging and fitness across a decade

Françoise Amélineau, David Grémillet, Ann MA Harding, Wojciech Walkusz, Rémi Choquet, Jérôme Fort

This file contains :

Supplementary Table S1.

Supplementary Figure S1.

Supplementary Figure S2.

Supplementary Methods.

Supplementary Table S1: Summary of time-depth recorders (TDRs) types, attachment methods and sample sizes

Year	TDR type	Attachment method	Recording rate	Number deployed	Number retrieved	Number analyzed	Reference
2004	LTD_1110, Lotek	Tesa® tape	5s	13	5	4	Harding et al 2009
2007	G5, Cefas	Loctite ®	5s	13	9	6	Grémillet et al 2012
2008	G5, Cefas	Intra-abdominal	2s	22	13	10	Fort et al 2010
2012	DST micro-TD, Star Oddi	Tesa® tape	4s	13	9	8	Amélineau et al 2016
2013	G5, Cefas	Tesa® tape	2s	16	8	8	this study
2014	G5, Cefas	Tesa® tape	2s	12	6	6	Amélineau et al 2016
2014	LUL, MIBE	Tesa® tape	1s	4	4	3	Amélineau et al 2016
2014	DST micro-TD, Star Oddi	Tesa® tape	4s	2	2	2	Amélineau et al 2016
2015	G5, Cefas	Tesa® tape	1s	21	20	20	this study

a.

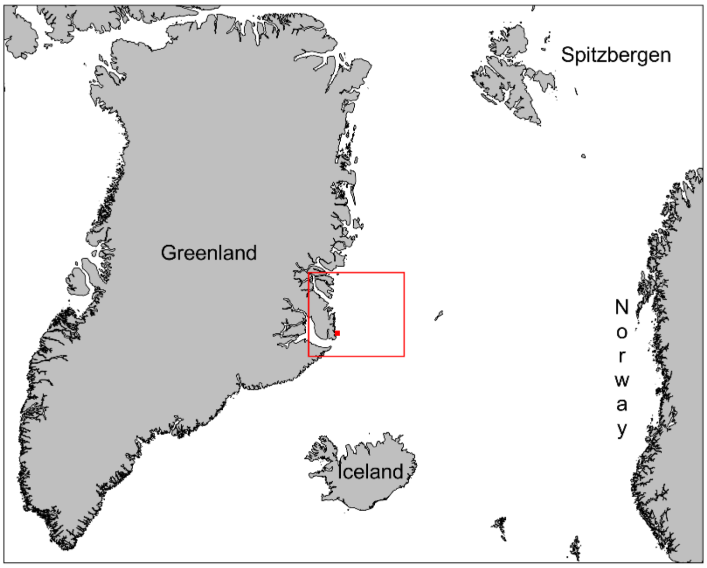

b.

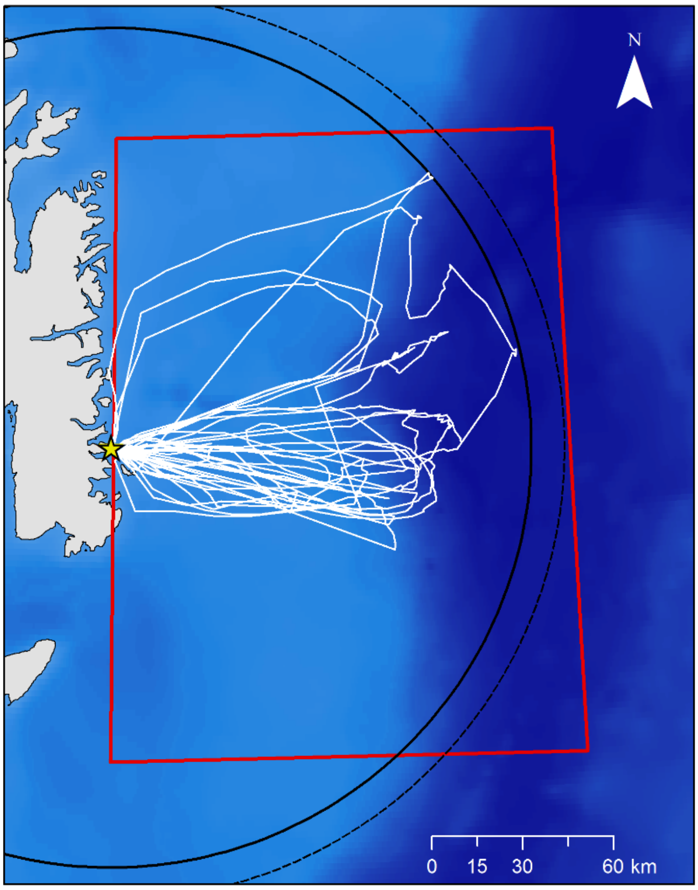

Supplementary Figure S1: a. location of the study site. b. Study site and GPS tracks of foraging birds in 2011, 2012 and 2014 (white lines); yellow star: colony; red polygon: area used for environmental parameters; black circle, bold line: maximum distance reached by a foraging little auk from our study site (139km); black circle, dashed line: maximum distance reached by a foraging little auk in Spitsbergen (150km, Jakubas et al. 2013, MEPS).

a. Bird LIAK15EG45, dive bout in the water column.

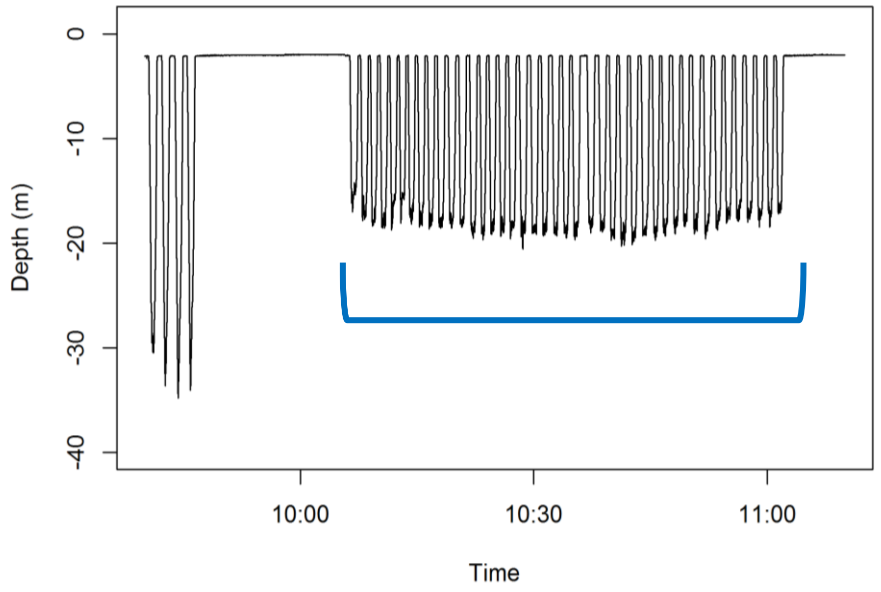

b. Bird LIAK15EG51, dive bout performed just under the sea-ice.

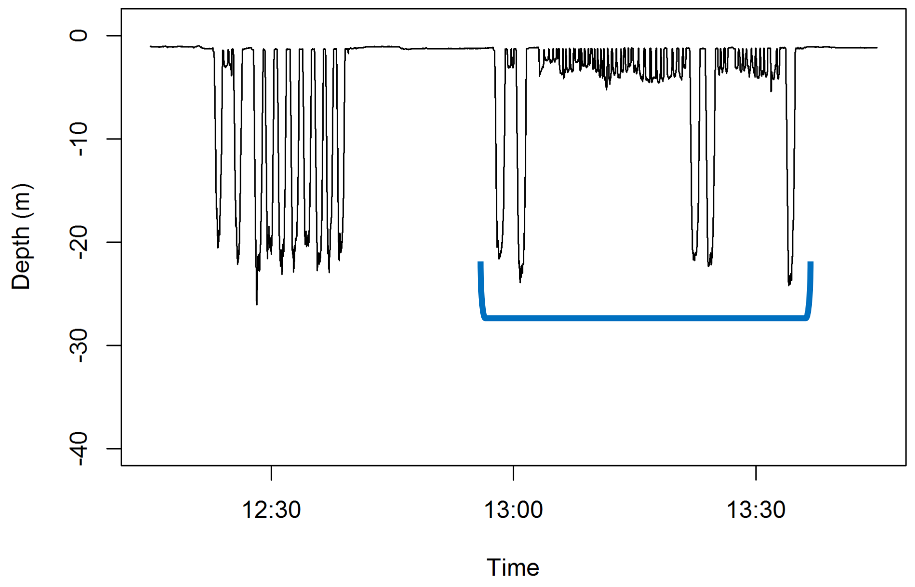

Supplementary Figure S2: Example of TDR recordings for dive bouts performed in the water column (a, bird LIAK15EG45), and under the sea-ice (b, bird LIAK15EG51).

Supplementary Methods

Temperature-depth recorders (TDRs) were deployed on chick-rearing adults. Breeding status was assessed by the presence of a full gular pouch and/or a breeding patch. Logger types are detailed in table S1 and were deployed ventrally with different attach methods. Loggers were attached directly on feathers with Tesa® tape or with Loctite® glue (2007 only). In 2008, birds were equipped with intra-abdominal TDRs (see details in Fort et al. 2010) and we could not measure time spent flying (based on temperature channel) for this year.
